# Hepatitis B- and Hepatitis C-Related Hepatocellular Carcinomas in the United States: Similarities and Differences

**DOI:** 10.5812/hepatmon.7635

**Published:** 2012-10-25

**Authors:** Jennifer Ng, Jennifer Wu

**Affiliations:** 1 Department of Medicine, New York University Langone Medical Center, New York, United State; 2 Division of Hematology and Medical Oncology, New York University Cancer Institute, New York, United State

**Keywords:** Hepatitis B Virus, Hepatitis C, Hepatocellular Carcinoma

## Abstract

**Context:**

Hepatitis B and Hepatitis C (HBV and HCV) infections are both major causes of hepatocellular carcinoma (HCC). However, HCC caused by each of these two viruses has unique characteristics that should be studied independently to that of another one. While HBV- and HCV-related HCCs share similar host and environmental risk factors such as male gender, age above 50 years old, family history of HCC, cirrhosis, obesity, and concomitant alcohol/tobacco use, they differ in their viral risk factors.

**Evidence Acquisition:**

The actual level of HBV DNA, the presence of HBV e antigen (HBeAg), and mutations in the viral genome are important predisposing factors to HCC development in HBV, whereas in HCV, viremia of any amount denotes an elevated risk. HBV and HCV also differ in their mechanisms of carcinogenesis. For example, HBV can integrate into the host genome and induce many different genetic alterations/mutations. Ultimately, though, both viruses act on similar pathways to produce HCC.

**Result:**

HBV and HCV are often transmitted differently - vertically (HBV) and horizontally (HCV), which may play a role in their distinct clinical presentations: HBV patients are younger and more frequently have larger/ bilobar tumors as opposed to HCV patients, who have worse liver function on diagnosis of HCC. Even the way they respond to treatment seems to be different. HBV-related HCC patients tend to progress faster after sorafenib treatments.

**Conclusions:**

Future studies should investigate the ways in which these differences between HBV- and HCV-related HCC can translate into more tailored treatment strategies for each etiology of HCC in order to improve outcomes of both.

## 1. Context

As the ninth leading cause of cancer-related deaths in the U.S., hepatocellular carcinoma (HCC) is a major cause of morbidity and mortality (1). The hepatitis viruses B and C (HBV and HCV) together are the most common etiologies of HCC and these two viruses are often categorized together as one risk factor in HCC studies. However, the two viruses confer different risks in the development of HCC; prevention and treatment of HCC caused by these two viruses, therefore, require different approaches. Thus, other than the fact that both belong to the family of hepatitis viruses, HBV and HCV are actually very different entities that should be studied separately in their relation to HCC. This review focuses on examining the similarities and differences between HBV- and HCV-related HCC in the US.

## 2. Evidence Acquisition

### 2.1. Epidemiology

Worldwide, the prevalence of HCC is estimated to be about 180 million people and the incidence continues to rise, especially in the U.S (2, 3). Liver and intrahepatic bile duct cancers rank among the seven cancers in the U.S. that show increasing incidence rates from 1999-2008 (2). The average annual incidence rate of HCC was 3.0 per 100,000 people between 2001-2006 in the U.S. (1). It continues to rise annually. Of note, incidence rates of HCC differ by race: Asians and Pacific Islanders carry the highest incidence of HCC, which corresponds to an incidence of 7.8 per 100,000 persons, while Caucasians are currently the least affected group, with an incidence of 2.6 per 100,000 persons (Table 1), though incidence rates are increasing among Caucasians and African Americans.

**Table 1:** Incidence Rates of HCC in the U.S. From 2001-2006

	Incidence Rate Per 100,000 Persons
**White**	2.6
**Black**	4.2
**American Indian/Alaskan Native**	3.2
**Asian/Pacific Islander**	7.8

Source: CDC’s Morbidity and Mortality Weekly Report (O’Connor S, Ward JW, Watson M, et al. Hepatocellular Carcinoma - United States, 2001-2006. CDC Morbidity and Mortality Weekly Report 2010; 59: 517-520)

The majority of HCC arises from viral hepatitis. In the U.S., 16% of HCC are attributed to HBV and 48% to HCV (2). Among HBV patients in North America, the incidence of HCC is 470 per 100,000 persons (4). The lifetime risk of HCC is 10-25% in chronically infected HBV patients. HCV patients, on the other hand, develop HCC at an annual rate of 1-4% (5). Chronic HCV infection occurs in 75-85% of HCV patients as opposed to only 5-10% of adults, 25-50% of children, and 90% of infants infected by HBV (6, 7). HCV confers a 17-fold increase in the risk for HCC (2). Similar to HBV-related HCC, most HCV-related HCC cases also tend to occur 25-30 years after chronic infection.

### 2.2. Risk of HCC Development

#### 2.2.1. Hepatitis B

##### 2.2.1.1. Modes of transmission and Genotype Variation

HBV is typically transmitted vertically, i.e. from mother to newborn or among siblings at younger ages in endemic areas such as Asia and Africa. In low-risk areas, horizontal transmission, i.e. via sexual and parenteral routes, is more the norm in the adulthood (3). Genotypes also vary from region to region and by ethnicity (Table 2). In the U.S., genotypes A and D are often seen in Caucasians and African Americans, as opposed to B and C in Asian Americans. Genotypes C, especially C2, and D are known to have a greater association with hepatocarcinogenesis (8), possibly because they are more likely to be found in patients with more severe liver disease (3). Of note, genotype B has been shown in some studies to be associated with the development of HCC in young non-cirrhotic carriers of HBV.

**Table 2:** HBV/HCV Genotypes and HCC

Genotype of HBV	Geographic Distribution	Genotype of HCV	Geographic Distribution
**A and D**	Africa, Europe, India, U.S.	1, 2, 3	U.S., Europe, Australia, East Asia
**B and C**	Asia, U.S.	4	Middle East, Egypt, Central Africa
**E**	West Africa	5	South Africa
**F**	Central and South America	6	South East Asia

Sources: El-Serag, H.B. Epidemiology of Viral Hepatitis and Hepatocellular Carcinoma. Gastroenterology 2012; 142: 1264-1273. Lee CM, Hung CH, Lu SN, et al. Hepatitis C virus genotypes: clinical relevance and therapeutic implications. Chang Gung Med J 2008; 31: 16-25.

##### 2.2.1.2. Overview of Risk Factors for HCC Development

Not all patients infected with viral hepatitis have the same risk of developing HCC. Risk factors associated with the development of HCC in chronically infected HBV patients can be divided into three categories - host, viral, and environmental factors (3).

### 2.3. Host

Host factors include male gender, age above 50 years old, family history of HCC, cirrhosis, and obesity (3, 8). Of note, all of these factors are also risk factors for HCV-related HCC. Multiple studies have shown that men are more likely to develop HCC than women, even after accounting for the greater incidence of alcoholic cirrhosis and viral hepatitis among men (3). There are several possible explanations to this fact. In studies on transgenic mice, transcription of HBV genes was increased by the androgen pathway (9, 10). Additionally, elevated testosterone levels correlate with increased risk of HCC (11). Finally, some studies have also shown a possible protective effect of estrogen (3). The ways in which age, obesity, and cirrho-sis serve as risk factors in viral hepatitis related HCC, however, are not well understood. We know that the longer a person is infected by hepatitis B, the higher the risk of development of HCC. One can speculate that advancing age heralds a less robust immune system, causing weaker host defenses against the hepatitis viruses and their carcinogenic effects. As for obesity, it increases the risk of hepatic steatosis (5) which can then lead to fibrosis and, ultimately, cirrhosis and HCC. Cirrhosis, while by itself is an independent risk factor for HCC and a by-product of viral hepatitis infection, presumably helps to provide a favorable background of liver damage, genetic mutations, and altered host immune responses for development of HCC in reaction to HBV and HCV.

### 2.4. Virus

Viral factors also contribute to the risk of HCC development. Elevated HBV DNA levels and presence of hepatitis B e antigen (HBe Ag) are both markers of active replication of HBV and risk factors for HCC (12). The REVEAL-HBV study from Taiwan looked at 3653 patients and found that the risk of HCC started to increase significantly at elevated serum HBV DNA level of 10,000 copies/ml. The higher the level of serum HBV DNA level, the higher the risk of HCC (Table 3). This dose-response relationship remained significant even in the absence of some indicators for hepatitis B severity such as HBeAg, ALT levels, and cirrhosis. Given these results, investigators have studied the impact of HBV treatment on the development/prevention of HCC. Studies have yielded conflicting results on the response of HBV to interferon treatment, though treatment with nucleoside analogs appears to be beneficial (8). One prospective randomized controlled trial with 651 patients showed a significantly lower risk of HCC development in the group who received lamivudine (a nucleoside analog) compared to placebo (3.9% vs. 7.4%, HR: 0.49, P = 0.047) (13). Similarly, a meta-analysis in 2008 involving 5 studies and 2289 patients showed a reduction in the risk of HCC by 78% (RR: 0.22, 95% CI: 0.10-0.50) in the group that received nucleoside analogs as compared to that of control group (14). Obviously, prevention is more effective than the treatment. With the advent of HBV vaccination, the overall incidence of HBV-related HCC has declined, by as much as 50% in certain areas such as Taiwan, proving that indeed, there is a direct causal relationship between HBV and HCC.

**Table 3:** Hazard Ratio for the Development of HCC by Serum HBV DNA Levels a

Serum HBV DNA level (copies/mL)	HR	95% CI	*P* value b
**300-9999**	1.4	0.5-3.8	0.56
**10,000-99,999**	4.5	1.8-11.4	0.001
**100,000-999,999**	11.3	4.5-28.4	< 0.001
**≥ 1 million**	17.7	6.8-46.3	< 0.001

Source: Chen CJ, Yang HI, Su J, et al. Risk of Hepatocellular Carcinoma Across a Biological Gradient of Serum Hepatitis B Virus DNA Level. JAMA 2006; 295: 65-73.

^a^HR values are based on comparison with participants with serum HBV DNA levels of less than 300 copies/mL. These results are from an analysis of a subset of 2925 participants seronegative for HBeAg, with normal ALT levels and no liver cirrhosis.

^b^P value < 0.05 is statistically significant

Other viral risk factors include mutations in the viral genome (15). The basal core promoter A1762T/G1764A mutation is considered as one of such mutations. It increases the risk of HCC by leading to enhanced viral replication, increased host immune response (causing further liver injury), and alterations in the coding region for the X antigen, which can interfere with cell growth control and DNA repairing. On the other hand, the precore G1896A mutation is associated with decreased risk of HCC. One explanation proposes that the wild-type precore, compared to the G1896A mutant, correlates with worse liver disease, which would lead to higher risk of HCC. This was shown in a cohort study in which patients infected by HBV with wild-type precore exhibited more hepatic inflammation and fibrosis than those with G1896A precore mutation (16). Lastly, other viral risk factors include genotypes C and D, as mentioned previously, as well as HDV, HIV or HCV co-infections (8).

### 2.5. Environmental

Environmental risk factors for HCC in HBV patients include concomitant heavy alcohol use, presumably by causing liver damage and cirrhosis, tobacco smoking - a known carcinogen, and exposure to aflatoxin B1(AFB1) (8). Aflatoxin B1 is a toxin produced by the fungi Aspergillus flavus and Aspergillus parasiticus that frequently grow on grains or groundnuts stored in moist conditions (3). It is a known hepatocarcinogen by itself, but also demonstrates synergistic effects with HBV to cause HCC. In an epidemiology study, the risk of HCC increased 60-fold among HBV carriers who showed AFB1 metabolites in their urine, compared to only a 4-fold increase among subjects who solely had AFB1 urinary metabolites without HBV, and a 7-fold increase in HBV infected subjects without AFB1 metabolites (17). Their synergistic effects can be explained by the fact that they both induce oxidative stress which predisposes to HCC (18). In addition, cells transfected by HBx protein are more susceptible to carcinogenic effects of AFB1 (19).

### 2.6. Hepatitis C

#### 2.6.1. Mode of Transmission

Hepatitis C is transmitted mainly via exposure to contaminated blood, either through drug abusers’ injection or in the health care setting (20). Other routes of transmission, i.e. perinatal or sexual, are less well understood. The rate of mother- child transmission in each pregnancy ranges from 4-7% in individuals with detectable maternal serum HCV RNA levels at the time of delivery (21). On the other hand, data are inconsistent on the circumstances under which HCV is sexually transmitted.

#### 2.6.2. Overview of Risk Factors for the Development of HCC

1) Host and Environment

Many of risk factors are common in both HBV- and HCV-related HCC, including age, male gender, family history of HCC, presence of cirrhosis, obesity, co-infection with HIV or HBV, and concomitant alcohol/tobacco use. Other risk factors for HCV-related HCC are advanced fibrosis, and possibly diabetes, increased liver iron stores, genotype 1b, and elevated HCV RNA levels (8).

With advancing stage of fibrosis or the increasing rate of fibrosis progression, the risk of HCC increases (3, 22). This may be explained by the rise in transaminase levels as fibrosis progresses, reflecting the increase of inflammation and oxidative DNA damage. On the other hand, the data on diabetes and increased liver iron stores are more controversial. Diabetic patients are more likely to develop non-alcoholic induced steatohepatitis which may subsequently progress to cirrhosis (5). Additionally, increased levels of insulin-like factors, which can be carcinogenic, are found in diabetic patients (5). Some studies from Europe have suggested that diabetes and HCV reveal synergistic effects in promoting the development of HCC but more data are needed for a conclusive answer (23, 24). Likewise, more data are required on excess liver iron stores as a possible risk factor of HCV-related HCC (22).

2) Virus

Genotype 1b and HCV RNA levels in the serum are well established risk factors. In large cohort studies and a recent meta-analysis, genotype 1b and higher titers of HCV RNA correlated with the risk of HCC in both cirrhotic and non-cirrhotic patients (22). Genotype 1b is associated with certain mutations in the core gene of HCV genome which promote HCC development, interferon treatment failure, and insulin resistance. Genotype 1b and genotype 1a are the most common genotypes in the U.S. and Europe (Table 2).

HCV viremia of any level is associated with the risk of HCC development (3), diffrent from HBV-related HCC in which the risk is statistically significant only when the viremia reaches to a certain level. On the other hand, similar to HBV-related HCC, the risk of HCV-related HCC can be reduced by the treatment of HCV disease. In a recent meta-analysis of 20 studies, the relative risk of HCC development in patients treated by either interferon or the combination of interferon and ribavirin was 0.43 compared to untreated patients (8). The meta-analysis also showed a relative risk of 0.35 for the occurrence of HCC in patients who demonstrated sustained virologic response (SVR) compared to non-responders. Of note, a sustained virologic response is defined as undetectable serum levels of virus for at least 6 months after cessation of antiviral treatment.

### 2.7. Pathogenesis of HCC in HBV and HCV

#### 2.7.1. Insult to the Liver

**Figure 1:**
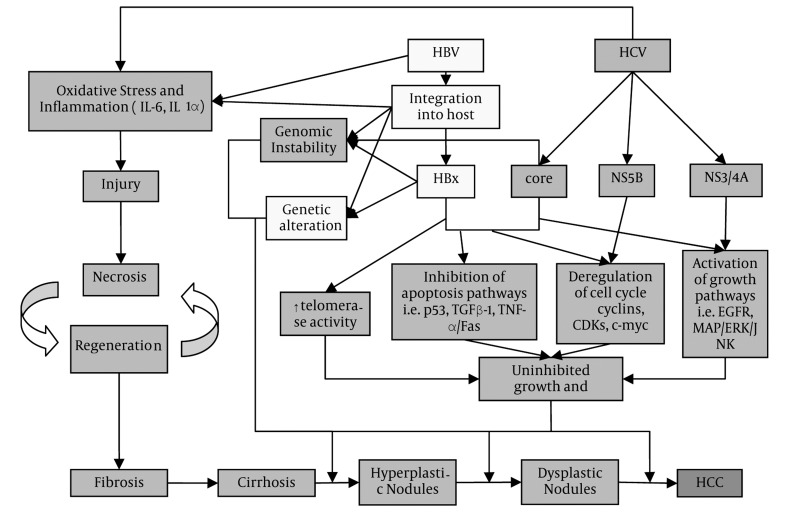
Pathogenesis of HCC in HBV and HCV Notes: Yellow = HBV related pathways, Blue = HCV related pathways, Green = common pathways, Red = end result

The development of HCC usually starts with injury to the liver (Figure 1). Both HBV and HCV are known to cause HCC via promoting inflammatory reactions and oxidative stress in the liver, though HCV is thought to contribute to greater oxidative DNA damage than HBV (8). Under these conditions, liver damage occurs, and sequentially followed by fibrosis, cirrhosis, and HCC (22).

HBV, a DNA virus, can also cause HCC in non-cirrhotic livers. HBV is known to integrate into the genomes of liver cells which can then contribute to HCC development in several different ways. The integration leads to rearrangements in the host DNA and possibly mutations in key regulatory cellular genes possibly providing a selective growth advantage in the targeted cells. In addition, cells with HBV-integrated DNA may express viral gene products which ultimately may cause tumorigenesis. For example, the integrated hepatitis B virus X (HBx) gene product induces chromosome instability by stimulating the development of an abnormal number of centrosomes, interfering with the mitotic checkpoint and inhibiting processes involved in sister chromatid separation during mitosis (25-27). Of note, the process of HBV DNA integration into the host genome also produces chromosomal instability, as well (8). Finally, less viral replication occurs after integration of HBV into the host cell genome, which helps infected cells evade the immune system as there is less HBV expression. Moreover, the defective HBV genomes produced after integration can also accumulate in the cell, causing the development of oxidative stress and DNA damage, leading to HCC development.

HCV, a RNA virus, cannot integrate into the host genome but it can also evade the host immune system and produce genomic instability like HBV (8). Its viral proteins can interfere with host defense mechanisms such as interferon (IFN) signaling pathway and the process of viral antigen presentation by MHC class I molecules, which then leads to chronic infection and eventually, malignant transformation (8, 22). The HCV core protein, similar to HBx protein, can also cause genomic instability via its interactions with the mitotic spindle cell checkpoint, thus contributing to the development of HCC (8).

#### 2.7.2. Cell Proliferation Pathways

HBV and HCV act on different points in similar molecular pathways in promoting growth and cell proliferation, an important step in hepatocarcinogenesis (8). The HBx protein in HBV raises mRNA levels of the epidermal growth factor receptor (EGFR) gene, leading to increased activation, whereas the HCV NS3/4A protease produces the same result by enhancing ligand binding to EGFR via proteolytic cleavage of the tyrosine phosphatase T-cell protein (28, 29). Both HBx and HCV core proteins also activate Ras and Raf mitogen-activated protein (MAP) kinases, extracellular signal-regulated kinases (ERKs), and c-Jun terminal kinases (JNKs) involved in cell proliferation signal transduction pathways (8). HBx also acts directly on cellular promoters and enhancers involved in the regulation of genes in the cell growth i.e. activator protein 1 (AP-1), AP-2, and nuclear factor kappa-B (NF-κB).

#### 2.7.3. Cell Cycle Regulation

Cell-cycle regulation is also affected by viral proteins of the two viruses. HBx and the pre-S2 mutation in HBV as well as the HCV NS5B protein have inhibitory effects on tumor suppressor retinoblastoma (pRb) signaling, which then promotes progression of the cell to the next phase along the cell-cycle (8). HBx increases activities and levels of various G1 phase proteins and kinases such as p21, p27, cyclin D1, cyclin E, and cyclin-dependent kinase 4 (CDK4), while inhibits others such as p15 and 16, leading to cell-cycle progression (30, 31). HCV core protein produces a similar effect through its activation of similar G1 phase kinases, such as CDK2, and inhibition of p21 CDK inhibitor (30, 31). HBx also increases the stability of intracellular c-myc while HCV core activates c-myc expression, both of which contribute to deregulation of the cell cycle.

#### 2.7.4. Apoptosis Pathways

The viral proteins of HBV and HCV can also block apoptosis. By activating PI 3-kinase (PI3K), HBx inhibits transforming growth factor beta-1 (TGFβ-1)-induced apoptosis (8). HBx also blocks apoptosis via p53-mediated,tumor necrosis factor (TNF)-α/Fas-mediated, and caspase-independent cell death pathways via sequestration of p53, activation of NF-κB, and upregulation of serine protease inhibitor Kazal (32-34). Similarly, HCV core interferes with p53-induced apoptosis and caspase-dependent cell death, while NS5A activates the PI3K-Akt cell survival pathway and inhibits both caspase-dependent cell death and mammalian target of rapamycin (mTOR)-mediated apoptosis (8).

#### 2.7.5. Uninhibited Replication, Angiogenesis, Invasion, and Metastasis

HBV and HCV viral proteins also induce unlimited replication of infected cells and promote angiogenesis, tissue invasion, and metastasis, which all contribute to carcinogenesis. Telomere maintenance allows continued replication of cells without stopping; both HBV and HCV infected cells achieve this by different mechanisms (8). HBx upregulates telomerase reverse transcriptase (hTERT), which is a subunit of telomerase (the enzyme involved in telomere maintenance), while HCV core protein increases telomerase activity. Replicating cells also require access to blood supply, which is sustained via angiogenesis. HBx promotes angiogenesis via modulating hypoxia-induced angiogenesis and other mediators of angiogenesis, i.e. angiopoietin-2 (Ang2) and vascular endothelial growth factor (VEGF) (35, 36). Likewise, HCV core protein acts on similar angiogenesis pathways (8). Finally, HBx activates various matrix metalloproteinases (MMPs), which are crucial to the ability of HCC cells to invade and metastasize. HCV core protein, on the other hand, promotes the development of a migratory mesenchymal phenotype in HCC cells via inhibition of TGFβ-related tumor suppressive activity.

### 2.8. Clinical Features of HCC by Viral Etiology

#### 2.8.1. Hepatitis B

Patients with HBV tend to develop HCC at a younger age than HCV patients, sometimes up to a decade earlier (37, 38). In the study by Rabe et al, the median age of HCC presentation in HBV infected patients was 55 (range 31-76) vs. 66 (range 42-82) in HCV patients. This is not surprising, given the high rates of vertical transmission among HBV patients. Also, Barazani et al noted that patients with HBV, compared to those with HCV, were significantly more likely to present with larger tumors (mean tumor size 5.1 cm vs. 3.7 cm), and 56% (HBV) vs. 18% (HCV) presented with tumors larger than 5 cm (37). HBV patients also had significantly higher incidence of bilobar liver involvement (P = 0.03) and were less likely to meet the Milan criteria for liver transplantation (P = 0.0289) (Table 4).

**Table 4:** Milan Criteria for Orthotopic Liver Transplantation

1	Single nodule ≤ 5 cm in diameter or up to 3 separate lesions all < 3 cm
2	No proven vascular invasion
3	No nodal or distant metastases

Sources: Raphael SW, Zhang Y, Chen YX, et al. Hepatocellular carcinoma: Focus on different aspects of management. ISRN Oncology vol. 2012, Article ID 421673, 12 pages, 2012. doi:10.5402/2012/421673.

#### 2.8.2. Hepatitis C

HCV patients, however, have significantly more evidence of liver dysfunction as opposed to their HBV counterparts. Wong et al found that HCV patients, compared to HBV patients, have significantly greater rates of stigmata of portal hypertension/cirrhosis and worse liver function on presentation (39). This was evidenced by higher rates of ascites, encephalopathy, thrombocytopenia, increased AST/ALT ratio, and hypoalbuminemia. In the study, HCV patients also showed significantly worse Child-Pugh class as well as worse Okuda stage. Child-Pugh class is a measure of the severity of liver disease while the Okuda staging system reflects worsening prognosis with advancing stages and takes into account both tumor stage and functional status of the patient (40). The poorer liver function and worse portal hypertension/cirrhosis seen in HCV patients may reflect the fact that oncogenesis in HCV patients occurs almost always after the development of fibrosis, followed by cirrhosis, and finally HCC, illustrating the greater amount of liver damage that has to occur prior to hepatocarcinogenesis in HCV patients. This is also supported by the fact that a greater percentage of HCV-related HCC patients develop cirrhosis, as compared to their HBV counterparts.

## 3. Results

### 3.1. Treatment and Outcome

Given the differences between the two viral etiologies of HCC, the question remains -if there is any difference in survival and response to the treatment among the two groups. At this point, there is paucity of data that compare survival of HBV- and HCV-related HCC among different treatment options, although the results are conflicting.

#### 3.1.1. Hepatic Resection

Hepatic resection and liver transplantation are both considered as curative therapies (40). Among these therapies, hepatic resection is the preferred option given the scarcity of livers available for transplant. In a Japanese study, 469 patients (14% HBV, 75% HCV) received curative hepatic resection and were followed up to 11 years post-operatively (41). The disease-free survival (DFS) rates at 3-, 5- and 10-years for HCV-related HCC group were significantly lower than that of HBV-related HCC group (40%, 24%, and 12% vs. 57%, 54%, and 28%, respectively). Overall survival (OS) between the two groups was not significantly different though 10 years after surgery, HCV-related HCC patients tended to have lower survival rates than those with HBV-related HCC. Wu et al, however, noted no significant difference in DFS rates, though OS of HBV-related HCC patients, who had undergone liver resection, was observed to be significantly lower than that of HCV-related HCC patients (42). However, this study composed of lower number of patients and shorter follow-up period, and was limited by selection bias. A recent meta-analysis of 14 studies on post-operative survival showed that both 5-year OS and DFS rates between surgically resected hepatitis B- and hepatitis C-related HCC groups were not significantly different (43). This meta-analysis included studies from Asia, Europe and the U.S., and length of follow-up was at least 5 years. It is unclear whether or not differences in ethnic groups and length of follow-up may play a role in explaining the conflicting results found among the various studies. Finally, while there are few studies comparing the effect of viral type on outcomes following hepatic resection, there are even less data on that of liver transplantation or radiofrequency ablation and they remain interesting avenues for further investigation.

#### 3.1.2. TACE/TAE

Trans-arterial embolization and chemoembolization (TAE and TACE) are available as palliative therapies for patients who are not candidates for the aforementioned curative therapies, and can serve as bridges to transplant (40). These two treatments involve the injection of certain substances into the arterial system of the liver in order to block the blood supply to HCC, with the option of adding chemotherapeutic agents as well, in the case of TACE. A retrospective case-control study looked at outcomes in non-surgical therapy for HCC, including TACE, after matching 102 HBV patients to 102 HCV patients by gender, age, and treatment center, period of enrollment, chronic liver disease, Child-Pugh class, HCC stage, and modality of HCC diagnosis. It showed no statistical difference in survival between the HBV- and HCV-related HCC groups with advanced HCC treated by radical procedures/TACE (44). However, extrapolating from a recent meta-analysis of TACE/TAE vs. conservative management/suboptimal therapies, there seems to be a suggestion that HBV-related HCC patients may do worse with TACE/TAE than HCV-related HCC patients (45). This meta-analysis looked at several trials, including 2 randomized controlled trials, one of which was composed of predominantly HBV-related HCC patients (80%) and the other, HCV-related HCC patients (85%) (46, 47). In the trial with predominantly HBV-related HCC, the survival rates at 1 and 2 years were 57% and 31%, respectively, as compared to 82% and 63%, respectively, in the trial with predominantly HCV-related HCC. Of note, cisplatin was used in the first study while doxorubicin in the second which might confound the results. No studies to date, however, have looked specifically at the potential difference between cisplatin and doxorubicin as chemo-embolization materials and most studies conclude that the specific chemotherapy component of chemo-embolization is not important, as embolization itself comprises the majority of the benefit of TACE in HCC treatment. Further study is needed to determine whether or not there is a difference in OS between HBV- and HCV-related HCC patients treated by TACE.

#### 3.1.3. Systemic Treatment With Sorafenib

Sorafenib is a multi-kinase inhibitor that blocks tumor proliferation and angiogenesis, and promotes apoptosis of tumor cells. It interferes with cellular signaling mediated by the serine-threonine kinase Raf-1 and VEGF pathways, two important pathways in the pathogenesis of HCC. The drug showed a survival benefit in advanced HCC in the multicenter, double-blind, placebo controlled, randomized control trial (RCT) SHARP (Sorafenib Hepatocellular Carcinoma Assessment Randomized Protocol) with 602 patients (48). SHARP showed a median OS of 10.7 months in the sorafenib group compared to 7.9 months in the placebo group. The survival benefit of sorafenib was then confirmed in Taiwan using the same study design, enrolling a total of 271 patients, demonstrating a median OS of 6.5 months and 4.2 months in the sorafenib and placebo groups, respectively (49). Even though all patients with advanced HCC derived benefits from sorafenib, Asian patients in Taiwan study, in overall showed worse outcomes. One observation pointed out to the different viral etiologies of HCC patients in two trials as a possible explanation. In the Asian study that followed SHARP trial, 89.7% of all viral hepatitis-related HCC was caused by hepatitis B, as compared to only 39.6% in SHARP trial. Instead, hepatitis C patients (60.4%) dominated the majority of viral hepatitis patients in SHARP trial while they only made up 10.3% of cases in Asian trial.

To investigate this observation, a retrospective analysis was performed on 46 patients (13 HCV+, 33 HBV+) in a phase II trial on HCC patients who received sorafenib, looking specifically for a difference between HBV versus HCV related HCC (50). While no statistical significant differences in OS and median progression free survival were found, there was a tendency towards a slower time to progression in HCV group vs. HBV group (6.5 versus 4 months, respectively). The findings suggest that sorafenib may be more efficacious in HCV-related HCC. To explain these findings, Huitzil-Melendez et al postulates that there may be significant differential expression of Raf-1, an important kinase involved in the oncogenesis of HCC, among HCV- and HBV-related HCC, given that HCV core proteins are known to induce a high basal rate of Raf-1 activity (50, 51). Through its highly effective inhibition of Raf-1 kinase, sorafenib may thereby slow down the progression of HCV-related HCC more efficaciously, as compared to that of HBV. In fact, an unplanned retrospective analysis of SHARP study showed that among the subset of patients with HCV-related HCC, the median OS was 14 months, much higher than 10.9 months of median OS for general population in the study (52). These 2 analyses were, however, limited by both being retrospective analyses and that both included very small number of HBV infected patients.

#### 3.1.4. Overall

The data on the effect of HBV and HCV infection on overall outcome of HCC patients are conflicting. One retrospective study looking at 255 HCC patients at a single center found that 5-year survival was 36% in HCV-related HCC patients, significantly lower than that of their HBV counterparts (56%) (37). However, the treatment modalities received by the patients in the study were not uniform. In addition, there were significantly less HCV-related HCC patients who had received surgical resection or chemoembolization which might explain significantly the lower OS of HCV-related HCC participants. In fact, when matched by gender, age, center, period of enrollment, chronic liver disease, Child-Pugh class, HCC stage, and modality of HCC diagnosis, HCV-related HCC patients tended to have better survival compared to their HBV counterparts. In fact, this difference in survival was actually statistically significant among patients with advanced HCC (hazard ratio 1.62, 95% CI: 1.06-2.48, P = 0.025) (44). Of note, non-advanced HCC was defined as solitary tumor < 5 cm or plurifocal tumors ≤ 3 lesions, with the largest diameter ≤ 3cm, without evidence of vascular invasion or distant metastases, while lesions that exceeded these criteria were defined as advanced HCC. The difference in survival among advanced HCC patients in two groups may be partially explained by the finding that in experimental animal models, a mutation in variant beta-estrogen receptors (beta-vER), which is more common in HBV-related HCC, occurs in the late stages of tumor progression, leading to a more aggressive tumor (53, 54).

## 4. Conclusion

While HBV- and HCV-related HCC share some similarities, they have many more differences, from the way they are transmitted, to their clinical presentations, and to most importantly, their response to treatment. Though HCV-related HCC patients appear to have worse liver function on presentation, their HBV counterparts tend to be less responsive to treatment and have worse outcomes in advanced HCC, at which point systemic therapy becomes the only treatment option. One possible explanation is that HBV can produce HCC without cirrhosis and the patient population is younger (with better liver reserve), the patients may not develop signs and symptoms of HCC until the tumor reaches a more aggressive state, when there are less treatment options available. Indeed, HBV-related HCC patients were significantly more likely to have larger and bilobar tumors, and significantly less likely to meet criteria for transplantation. This may drive HBV-related HCC patients away from curative treatment, toward palliative treatments such as TACE/TAE and sorafenib, which appears to be more suited for treatment of HCV-related HCC. Thus, the key to decreasing HBV-related HCC currently lies more in prevention rather than treatment of the cancer. In fact, lamivudine and other nucleoside analogs do show some promise in reducing the risk of HBV-related HCC by treating HBV itself. However, further investigation is required to determine if the benefit of nucleoside analogs is confirmed in future studies. As our understanding of the biological differences in viral hepatitis-related HCC progresses, there is a hope that in the future it will help guidance of the development of targeted therapies based on differences found between these viral etiologies. Ultimately, we hope to be able to improve the outcome of viral hepatitis-related HCC and to lessen the morbidity and mortality of this disease that attacks many individuals in the world.
